# Chlorido­bis­(1,10-phenanthroline-κ^2^ *N*,*N*′)copper(II) chlorido­(1,10-phen­anthroline-κ^2^ *N*,*N*′)(pyridine-2,6-di­carboxyl­ato-κ^3^ *O* ^2^,*N*,*O* ^6^)manganate(II) methanol monosolvate

**DOI:** 10.1107/S1600536814006369

**Published:** 2014-03-29

**Authors:** Halyna I. Buvailo, Julia A. Rusanova, Valeriya G. Makhankova, Vladimir N. Kokozay, Roman I. Zubatyuk

**Affiliations:** a Taras Shevchenko National University of Kyiv, Department of Inorganic Chemistry, Volodymyrska str. 64/13, 01601 Kyiv, Ukraine; b National Taras Shevchenko University of Kyiv, Department of Chemistry, Volodymyrska str. 64, 01033 Kyiv, Ukraine; c Institute for Scintillation Materials, "Institute for Single Crystals", National Academy of Sciences of Ukraine, Lenina ave. 60, Kharkov 61001, Ukraine

## Abstract

The title complex, [CuCl(C_12_H_8_N_2_)_2_][Mn(C_7_H_3_NO_4_)Cl(C_12_H_8_N_2_)]·CH_3_OH, consists of discrete [CuCl(phen)_2_]^+^ cations (phen is 1,10-phenanthroline), [MnCl(pydc)(phen)]^−^ anions (H_2_pydc is 2,6-pyridine-2,6-di­carb­oxy­lic acid) and one methanol solvent mol­ecule of crystallization per asymmetric unit. It should be noted, that a solvent-masking procedure as implemented in *OLEX2* [Dolomanov *et al.* (2009). *J. Appl. Cryst.* **42**, 339–341 ▶] was used to remove the electronic contribution from one disordered solvent molecule, presumably methanol. Only the atoms used in the refined model are reported in chemical formula and related values. The Cu^II^ ion is five-coordinated by two phenanthroline ligands and one chloride ion in a distorted trigonal–bipyramidal geometry. The dihedral angle between the phen ligands is 65.21 (5)°. The Mn^II^ ion is six-coordinated by one Cl atom, two N atoms from a phen ligand, as well one N atom and two O atoms from pydc in a distorted octa­hedral coordination geometry, with *cis* angles ranging from 72.00 (8) to 122.07 (8)° and *trans* angles ranging from 143.98 (8) to 163.15 (6)°. In the crystal, C—H⋯O, O—H⋯O and C—H⋯Cl hydrogen bonds, cation–anion π–π inter­actions between the phen ring systems with centroid–centroid distances in the range 3.881 (34)–4.123 (36) Å, as well as cation–cation, anion–anion π–π inter­actions between the phen rings with centroid–centroid distances in the range 3.763 (4)–3.99 (5) Å and pydc rings with centroid–centroid distances 3.52 (5) Å link the various components.

## Related literature   

For background to the direct synthesis of heterometallic complexes, see: Chygorin *et al.* (2012 ▶); Nesterov *et al.* (2012 ▶); Nesterova *et al.* (2013 ▶). For the structures of related complexes, see: Wei & Yang (2004 ▶); Lu *et al.* (2004 ▶); Murphy *et al.* (1997 ▶); Liu *et al.* (2006 ▶); Ma *et al.* (2002 ▶); Laine *et al.* (1995 ▶); Chatterjee *et al.* (1998 ▶).
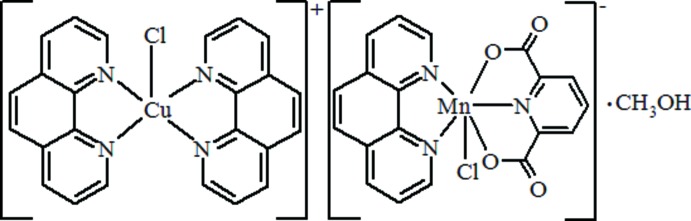


## Experimental   

### 

#### Crystal data  

[CuCl(C_12_H_8_N_2_)_2_][MnCl(C_7_H_3_NO_4_)Cl(C_12_H_8_N_2_)]·CH_4_O
*M* *_r_* = 927.14
Triclinic, 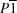

*a* = 10.3680 (4) Å
*b* = 12.5332 (4) Å
*c* = 17.2709 (6) Å
α = 72.966 (3)°
β = 74.709 (3)°
γ = 89.262 (3)°
*V* = 2064.78 (13) Å^3^
*Z* = 2
Mo *K*α radiation
μ = 1.01 mm^−1^
*T* = 293 K
0.25 × 0.19 × 0.11 mm

#### Data collection  

Oxford Diffraction Xcalibur Sapphire3 diffractometer
Absorption correction: analytical (Clark & Reid, 1995 ▶) *T*_min_ = 0.837, *T*_max_ = 0.918
35601 measured reflections
9973 independent reflections
6764 reflections with *I* > 2σ(*I*)
*R*_int_ = 0.040

#### Refinement  

*R*[*F* ^2^ > 2σ(*F* ^2^)] = 0.049
*wR*(*F* ^2^) = 0.140
*S* = 1.05
9973 reflections
543 parameters
H-atom parameters constrained
Δρ_max_ = 0.44 e Å^−3^
Δρ_min_ = −0.51 e Å^−3^

### 

Data collection: *CrysAlis PRO* (Oxford Diffraction, 2010 ▶); cell refinement: *CrysAlis PRO*; data reduction: *CrysAlis PRO*; program(s) used to solve structure: *SHELXTL* (Sheldrick, 2008 ▶); program(s) used to refine structure: *OLEX2* (Dolomanov *et al.*, 2009 ▶); molecular graphics: *SHELXTL*; software used to prepare material for publication: *publCIF* (Westrip, 2010 ▶).

## Supplementary Material

Crystal structure: contains datablock(s) I. DOI: 10.1107/S1600536814006369/br2237sup1.cif

Structure factors: contains datablock(s) I. DOI: 10.1107/S1600536814006369/br2237Isup2.hkl

CCDC reference: 993053

Additional supporting information:  crystallographic information; 3D view; checkCIF report

## Figures and Tables

**Table 1:** Hydrogen-bond geometry (Å, °)

*D*—H⋯*A*	*D*—H	H⋯*A*	*D*⋯*A*	*D*—H⋯*A*
C22—H22⋯Cl2^i^	0.93	2.87	3.683 (4)	147
C34—H34⋯Cl2	0.93	2.88	3.492 (3)	124
C30—H30⋯Cl2^ii^	0.93	2.87	3.751 (3)	158
C44—H44*C*⋯Cl1^iii^	0.96	2.86	3.574 (6)	132
O5—H5*A*⋯O2	0.82	2.03	2.798 (5)	155
C20—H20⋯O3	0.93	2.45	3.337 (4)	159

Symmetry codes: (i) 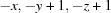; (ii) 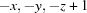; (iii) 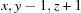.

**Fig. 1.**
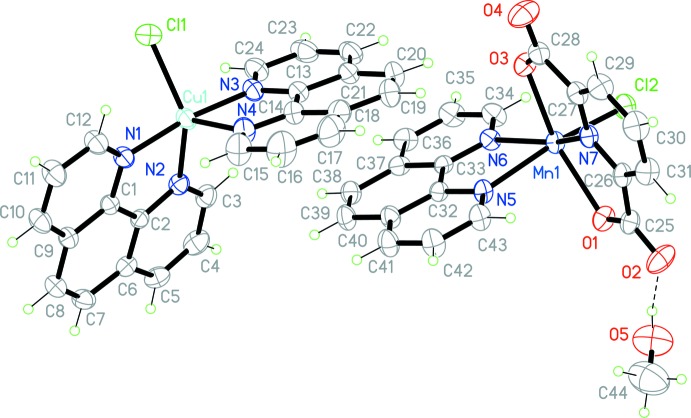
The molecular structure of the title compound with hydrogen bonds with solvent molecule shown as dashed lines.

**Fig. 2.**
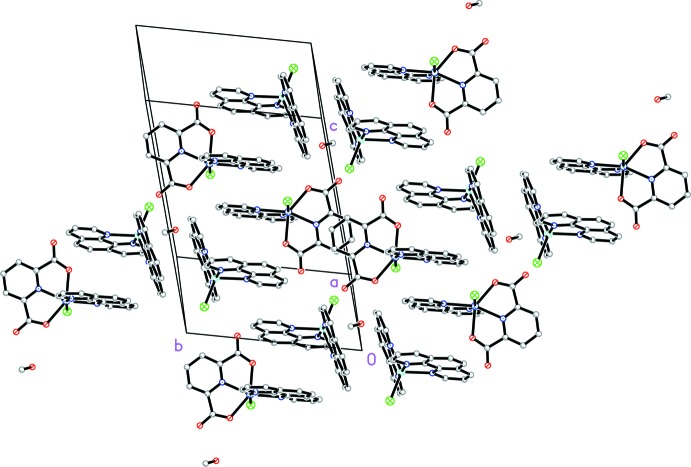
Part of the crystal structure showing cation–anion and cation–cation, anion–anion π–π interactions. H atoms are omited for clarity.

## supplementary crystallographic information

### 1. Comment

This work is a continuation of our research in the field of direct synthesis of heterometallic complexes (Chygorin *et al.*, 2012; Nesterov *et al.*, 2012; Nesterova *et al.*, 2013). In this paper we present a novel Cu/Mn heterometallic ionic complex with pyridine-2,6-dicarboxylic acid in combination with 1,10-phenanthroline as a ligands selected to construct supramolecular heterometallic assemblies.

As is shown in Fig. 1. the unit of the title compound consists of [CuCl(phen)_2_]^+^ cations, [MnCl(2,6-pydc)(phen)]^-^ anions and solvent (MeOH) molecule. The Cu^II^ ion adopts a distorted trigonal-bipyramidal environment by coordinating with four nitrogen atoms from two phen ligands and one Cl atom. The dihedral angle between the two phen ligands (114.79(0.05)°) as well as the range of Cu—N bond distances of 1.997 (2) - 2.174 (3) Å is in good agreement with the previously reported values for analagous complexes (Wei *et al.*, 2004; Lu *et al.*, 2004; Murphy *et al.*, 1997; Liu *et al.*, 2006). The Mn^II^ centre is surrounded by one bidentate phenanthroline ligand, one tridentate dipicolinate ligand and one chlorine atom and exhibits distorted octahedral geometry. The range of Mn—N and Mn—O bond distances of 2.173 (2) - 2.302 (2) Å and 2.257 (2) - 2.266 (2) Å respectively as well as *cis* angles ranging from 72.00 (8)° to 122.07 (8)° and *trans* angles ranging from 143.98 (8)° to 163.15 (6)° are in a good agreement with literature values (Ma *et al.*, 2002; Laine *et al.*, 1995; Chatterjee *et al.*, 1998).

In the crystal anions are inolved in the formation of C—H···O hydrogen bonds with solvent molecules and O—H···O and C—H···Cl hydrogen bonds with cations with D···A distances ranging from 2.798 (5) to 3.751 (3) Å. In addition, cation–anion π–π interactions between the phen ring systems with centroid–centroid distances in the range 3.88 (3)-4.12 (4) Å and cation–cation, anion–anion π–π interactions between the phen rings with centroid–centroid distances in the range 3.76 (4)–3.99 (5) Å and pydc rings with centroid–centroid distances 3.52 (5) Å connect the various components together.

### 2. Experimental

Copper powder (0.08 g, 1.25 mmol), KMnO_4_ (0.2 g, 1.25 mmol), phen·H_2_O (0.50 g, 2.5 mmol) and NH_4_Cl (0.14 g, 2.5 mmol) in CH_3_OH (20 mL) were mechanically stirred at 323–333 K in air until total dissolution of copper was observed (3 h). The resulting green solution was filtered from the insignificant quantities of by-products and cooled to the room temperature. The Pr^i^OH was added dropwise within 2 days to obtain green crystals of the title complex. Yield 0.23 g (40% by copper).

### 3. Refinement

All non-hydrogen atoms were refined isotropically. All hydrogen atoms were placed at calculated position and refined in a riding-model approximation. Idealised Me and tetrahedral OH refined as rotating groups. Refinement without SQUEEZE procedure clearly shows severely disordered isolated solvent molecule, presumably methanol. However, we were unable to find acceptable model of disorder.

### Crystal data

**Table:**

[CuCl(C_12_H_8_N_2_)_2_][MnCl(C_7_H_3_NO_4_)Cl(C_12_H_8_N_2_)]·CH_4_O	*Z* = 2
*M**_r_* = 927.14	*F*(000) = 944
Triclinic, *P*1	*D*_x_ = 1.491 Mg m^−^^3^
Hall symbol: -P 1	Mo *K*α radiation, λ = 0.71073 Å
*a* = 10.3680 (4) Å	Cell parameters from 6590 reflections
*b* = 12.5332 (4) Å	θ = 2.4–27.2°
*c* = 17.2709 (6) Å	µ = 1.01 mm^−^^1^
α = 72.966 (3)°	*T* = 293 K
β = 74.709 (3)°	Block, clear light green
γ = 89.262 (3)°	0.25 × 0.19 × 0.11 mm
*V* = 2064.78 (13) Å^3^	

### Data collection

**Table:**

Oxford Diffraction Xcalibur Sapphire3 diffractometer	9973 independent reflections
Radiation source: Enhance (Mo) X-ray Source	6764 reflections with *I* > 2σ(*I*)
Graphite monochromator	*R*_int_ = 0.040
Detector resolution: 16.1827 pixels mm^-1^	θ_max_ = 28.0°, θ_min_ = 2.0°
ω and π scans	*h* = −15→15
Absorption correction: analytical (Clark & Reid, 1995)	*k* = −18→18
*T*_min_ = 0.837, *T*_max_ = 0.918	*l* = −26→24
35601 measured reflections	

### Refinement

**Table:**

Refinement on *F*^2^	0 restraints
Least-squares matrix: full	Hydrogen site location: inferred from neighbouring sites
*R*[*F*^2^ > 2σ(*F*^2^)] = 0.049	H-atom parameters constrained
*wR*(*F*^2^) = 0.140	*w* = 1/[σ^2^(*F*_o_^2^) + (0.0706*P*)^2^ + 0.2578*P*] where *P* = (*F*_o_^2^ + 2*F*_c_^2^)/3
*S* = 1.05	(Δ/σ)_max_ = 0.001
9973 reflections	Δρ_max_ = 0.44 e Å^−^^3^
543 parameters	Δρ_min_ = −0.51 e Å^−^^3^

### Special details

**Table:**

Geometry. All e.s.d.'s (except the e.s.d. in the dihedral angle between two l.s. planes) are estimated using the full covariance matrix. The cell e.s.d.'s are taken into account individually in the estimation of e.s.d.'s in distances, angles and torsion angles; correlations between e.s.d.'s in cell parameters are only used when they are defined by crystal symmetry. An approximate (isotropic) treatment of cell e.s.d.'s is used for estimating e.s.d.'s involving l.s. planes.

### Fractional atomic coordinates and isotropic or equivalent isotropic displacement parameters (Å^2^)

**Table:**

	*x*	*y*	*z*	*U*_iso_*/*U*_eq_	
Mn1	0.20341 (4)	0.29043 (3)	0.50838 (3)	0.03953 (12)	
Cu1	0.72013 (3)	0.78783 (3)	0.01669 (2)	0.04942 (12)	
Cl1	0.61596 (9)	0.88250 (8)	−0.08120 (6)	0.0714 (3)	
Cl2	0.00242 (8)	0.27997 (7)	0.62072 (5)	0.0585 (2)	
O1	0.2901 (2)	0.15048 (16)	0.59210 (13)	0.0523 (5)	
O2	0.3165 (3)	−0.03177 (19)	0.62979 (16)	0.0783 (8)	
O3	0.1150 (2)	0.33570 (17)	0.39765 (12)	0.0512 (5)	
O4	0.0585 (3)	0.2738 (2)	0.30277 (17)	0.0837 (8)	
O5	0.4459 (4)	0.0292 (4)	0.7352 (3)	0.1196 (12)	
H5A	0.3971	0.0297	0.7047	0.179*	
N1	0.9021 (2)	0.8129 (2)	−0.06475 (14)	0.0462 (6)	
N2	0.8308 (2)	0.8280 (2)	0.09067 (14)	0.0448 (5)	
N3	0.5495 (2)	0.7495 (2)	0.10901 (16)	0.0520 (6)	
N4	0.6926 (3)	0.6098 (2)	0.03421 (18)	0.0630 (7)	
N7	0.1860 (2)	0.14053 (18)	0.47194 (14)	0.0408 (5)	
N5	0.4197 (2)	0.32798 (18)	0.42308 (15)	0.0430 (5)	
N6	0.2590 (2)	0.47013 (18)	0.48676 (14)	0.0423 (5)	
C1	1.0019 (3)	0.8371 (2)	−0.03370 (17)	0.0403 (6)	
C2	0.9630 (3)	0.8457 (2)	0.05011 (16)	0.0397 (6)	
C3	0.7928 (3)	0.8376 (3)	0.16789 (18)	0.0533 (7)	
H3	0.7021	0.8262	0.1963	0.064*	
C4	0.8819 (4)	0.8638 (3)	0.2084 (2)	0.0649 (9)	
H4	0.8511	0.8698	0.2625	0.078*	
C5	1.0152 (4)	0.8804 (3)	0.1673 (2)	0.0593 (8)	
H5	1.0763	0.8966	0.1940	0.071*	
C6	1.0604 (3)	0.8733 (2)	0.08529 (18)	0.0459 (7)	
C7	1.1967 (3)	0.8916 (3)	0.0360 (2)	0.0555 (8)	
H7	1.2619	0.9103	0.0588	0.067*	
C8	1.2337 (3)	0.8827 (3)	−0.0427 (2)	0.0526 (7)	
H8	1.3236	0.8944	−0.0729	0.063*	
C9	1.1356 (3)	0.8554 (2)	−0.08018 (18)	0.0449 (6)	
C10	1.1656 (3)	0.8471 (3)	−0.1617 (2)	0.0585 (8)	
H10	1.2540	0.8575	−0.1947	0.070*	
C11	1.0657 (3)	0.8237 (3)	−0.1927 (2)	0.0636 (9)	
H11	1.0852	0.8195	−0.2474	0.076*	
C12	0.9348 (3)	0.8060 (3)	−0.1429 (2)	0.0590 (8)	
H12	0.8675	0.7888	−0.1647	0.071*	
C13	0.5034 (3)	0.6408 (3)	0.1362 (2)	0.0540 (8)	
C14	0.5776 (3)	0.5656 (3)	0.0954 (2)	0.0566 (8)	
C15	0.7615 (5)	0.5422 (3)	−0.0057 (3)	0.0823 (12)	
H15	0.8415	0.5705	−0.0466	0.099*	
C16	0.7186 (6)	0.4310 (4)	0.0114 (3)	0.1014 (15)	
H16	0.7683	0.3868	−0.0188	0.122*	
C17	0.6042 (5)	0.3875 (4)	0.0722 (3)	0.0942 (14)	
H17	0.5753	0.3131	0.0839	0.113*	
C18	0.5297 (4)	0.4535 (3)	0.1174 (3)	0.0728 (10)	
C19	0.4089 (4)	0.4157 (4)	0.1834 (3)	0.0880 (14)	
H19	0.3766	0.3414	0.1992	0.106*	
C20	0.3403 (4)	0.4854 (4)	0.2233 (3)	0.0819 (13)	
H20	0.2625	0.4576	0.2665	0.098*	
C21	0.3841 (3)	0.6008 (3)	0.2010 (2)	0.0624 (9)	
C22	0.3149 (3)	0.6773 (4)	0.2377 (2)	0.0698 (10)	
H22	0.2365	0.6541	0.2811	0.084*	
C23	0.3627 (3)	0.7868 (4)	0.2095 (2)	0.0712 (10)	
H23	0.3172	0.8387	0.2336	0.085*	
C24	0.4799 (3)	0.8202 (3)	0.1446 (2)	0.0607 (8)	
H24	0.5107	0.8952	0.1253	0.073*	
C25	0.2817 (3)	0.0541 (2)	0.58579 (18)	0.0488 (7)	
C26	0.2221 (3)	0.0444 (2)	0.51651 (18)	0.0453 (6)	
C27	0.1351 (3)	0.1451 (2)	0.40829 (18)	0.0471 (7)	
C28	0.0989 (3)	0.2606 (3)	0.36521 (19)	0.0500 (7)	
C29	0.1164 (3)	0.0505 (3)	0.3861 (2)	0.0616 (9)	
H29	0.0815	0.0544	0.3410	0.074*	
C30	0.1507 (4)	−0.0504 (3)	0.4324 (2)	0.0682 (10)	
H30	0.1369	−0.1156	0.4195	0.082*	
C31	0.2059 (4)	−0.0542 (3)	0.4980 (2)	0.0605 (8)	
H31	0.2314	−0.1212	0.5288	0.073*	
C32	0.4672 (3)	0.4359 (2)	0.39983 (16)	0.0391 (6)	
C33	0.3820 (3)	0.5109 (2)	0.43562 (16)	0.0374 (6)	
C34	0.1806 (3)	0.5398 (3)	0.5192 (2)	0.0559 (8)	
H34	0.0954	0.5130	0.5541	0.067*	
C35	0.2213 (3)	0.6513 (3)	0.5028 (2)	0.0601 (9)	
H35	0.1634	0.6976	0.5265	0.072*	
C36	0.3439 (3)	0.6924 (2)	0.4529 (2)	0.0535 (8)	
H36	0.3719	0.7666	0.4428	0.064*	
C37	0.4295 (3)	0.6222 (2)	0.41604 (19)	0.0440 (6)	
C38	0.5602 (3)	0.6602 (3)	0.3616 (2)	0.0535 (8)	
H38	0.5915	0.7341	0.3494	0.064*	
C39	0.6397 (3)	0.5895 (2)	0.3272 (2)	0.0562 (8)	
H39	0.7245	0.6157	0.2915	0.067*	
C40	0.5944 (3)	0.4756 (2)	0.34522 (19)	0.0497 (7)	
C41	0.6731 (3)	0.3983 (3)	0.3127 (2)	0.0627 (9)	
H41	0.7574	0.4211	0.2751	0.075*	
C42	0.6249 (3)	0.2899 (3)	0.3368 (2)	0.0638 (9)	
H42	0.6763	0.2376	0.3165	0.077*	
C43	0.4988 (3)	0.2581 (2)	0.3919 (2)	0.0537 (8)	
H43	0.4677	0.1835	0.4079	0.064*	
C44	0.5429 (6)	−0.0325 (7)	0.7194 (4)	0.148 (3)	
H44A	0.5849	−0.0104	0.6599	0.221*	
H44B	0.5091	−0.1093	0.7380	0.221*	
H44C	0.6074	−0.0238	0.7484	0.221*	

### Atomic displacement parameters (Å^2^)

**Table:**

	*U*^11^	*U*^22^	*U*^33^	*U*^12^	*U*^13^	*U*^23^
Mn1	0.0478 (2)	0.0291 (2)	0.0440 (2)	−0.00048 (16)	−0.00924 (18)	−0.01727 (17)
Cu1	0.03775 (19)	0.0562 (2)	0.0496 (2)	−0.00171 (15)	−0.00515 (15)	−0.01451 (17)
Cl1	0.0576 (5)	0.0819 (6)	0.0751 (6)	0.0079 (4)	−0.0284 (4)	−0.0149 (5)
Cl2	0.0575 (4)	0.0610 (5)	0.0521 (4)	0.0001 (4)	−0.0054 (3)	−0.0181 (4)
O1	0.0729 (14)	0.0371 (11)	0.0513 (12)	0.0012 (9)	−0.0191 (10)	−0.0178 (9)
O2	0.126 (2)	0.0424 (13)	0.0748 (17)	0.0173 (13)	−0.0472 (16)	−0.0138 (12)
O3	0.0658 (13)	0.0420 (11)	0.0494 (12)	0.0049 (9)	−0.0185 (10)	−0.0165 (9)
O4	0.126 (2)	0.0696 (17)	0.0788 (18)	0.0079 (15)	−0.0622 (18)	−0.0267 (14)
O5	0.116 (3)	0.146 (3)	0.131 (3)	0.036 (2)	−0.051 (2)	−0.078 (3)
N1	0.0421 (12)	0.0580 (15)	0.0396 (13)	−0.0029 (11)	−0.0092 (10)	−0.0175 (11)
N2	0.0468 (13)	0.0479 (14)	0.0378 (12)	0.0068 (10)	−0.0078 (10)	−0.0137 (10)
N3	0.0415 (13)	0.0549 (16)	0.0535 (15)	0.0003 (11)	−0.0063 (11)	−0.0127 (12)
N4	0.0590 (16)	0.0509 (16)	0.0680 (18)	0.0048 (13)	−0.0024 (14)	−0.0145 (14)
N7	0.0461 (12)	0.0333 (12)	0.0428 (13)	−0.0028 (9)	−0.0065 (10)	−0.0159 (10)
N5	0.0486 (13)	0.0301 (11)	0.0484 (13)	0.0012 (9)	−0.0036 (10)	−0.0172 (10)
N6	0.0444 (13)	0.0362 (12)	0.0511 (14)	0.0032 (10)	−0.0119 (11)	−0.0214 (10)
C1	0.0395 (14)	0.0398 (15)	0.0412 (15)	0.0024 (11)	−0.0097 (11)	−0.0124 (12)
C2	0.0419 (14)	0.0368 (14)	0.0376 (14)	0.0036 (11)	−0.0106 (11)	−0.0071 (11)
C3	0.0567 (18)	0.0568 (19)	0.0394 (16)	0.0072 (14)	−0.0054 (13)	−0.0107 (14)
C4	0.083 (3)	0.073 (2)	0.0423 (18)	0.0178 (19)	−0.0187 (17)	−0.0222 (16)
C5	0.070 (2)	0.067 (2)	0.0500 (18)	0.0124 (17)	−0.0298 (17)	−0.0186 (16)
C6	0.0527 (16)	0.0423 (16)	0.0436 (16)	0.0049 (12)	−0.0166 (13)	−0.0111 (12)
C7	0.0500 (17)	0.0566 (19)	0.066 (2)	0.0009 (14)	−0.0271 (15)	−0.0171 (16)
C8	0.0407 (15)	0.0582 (19)	0.0575 (19)	−0.0012 (13)	−0.0098 (13)	−0.0184 (15)
C9	0.0409 (14)	0.0471 (16)	0.0449 (16)	0.0006 (12)	−0.0069 (12)	−0.0151 (13)
C10	0.0427 (16)	0.076 (2)	0.0552 (19)	−0.0015 (15)	0.0000 (14)	−0.0283 (17)
C11	0.0574 (19)	0.089 (3)	0.0452 (18)	−0.0056 (17)	−0.0023 (15)	−0.0319 (18)
C12	0.0514 (18)	0.082 (2)	0.0484 (18)	−0.0056 (16)	−0.0119 (14)	−0.0277 (17)
C13	0.0430 (16)	0.061 (2)	0.0505 (18)	−0.0009 (14)	−0.0122 (13)	−0.0064 (15)
C14	0.0525 (18)	0.0520 (19)	0.0580 (19)	−0.0057 (14)	−0.0134 (15)	−0.0066 (15)
C15	0.089 (3)	0.063 (3)	0.082 (3)	0.008 (2)	0.001 (2)	−0.025 (2)
C16	0.119 (4)	0.062 (3)	0.116 (4)	0.016 (3)	−0.008 (3)	−0.038 (3)
C17	0.117 (4)	0.051 (2)	0.113 (4)	0.000 (2)	−0.033 (3)	−0.021 (2)
C18	0.074 (2)	0.057 (2)	0.082 (3)	−0.0050 (18)	−0.028 (2)	−0.0056 (19)
C19	0.074 (3)	0.065 (3)	0.103 (3)	−0.024 (2)	−0.023 (2)	0.008 (2)
C20	0.057 (2)	0.084 (3)	0.078 (3)	−0.021 (2)	−0.0114 (19)	0.011 (2)
C21	0.0422 (16)	0.080 (3)	0.0530 (19)	−0.0062 (16)	−0.0116 (14)	−0.0031 (17)
C22	0.0420 (17)	0.109 (3)	0.0502 (19)	−0.0015 (19)	−0.0060 (14)	−0.017 (2)
C23	0.0467 (18)	0.105 (3)	0.065 (2)	0.0156 (19)	−0.0124 (16)	−0.033 (2)
C24	0.0468 (17)	0.068 (2)	0.067 (2)	0.0076 (15)	−0.0092 (15)	−0.0243 (18)
C25	0.0603 (18)	0.0370 (16)	0.0460 (16)	−0.0001 (13)	−0.0102 (14)	−0.0113 (13)
C26	0.0525 (16)	0.0322 (14)	0.0483 (16)	−0.0031 (12)	−0.0044 (13)	−0.0156 (12)
C27	0.0487 (16)	0.0457 (16)	0.0505 (17)	−0.0043 (12)	−0.0094 (13)	−0.0233 (13)
C28	0.0524 (17)	0.0539 (19)	0.0455 (17)	−0.0035 (13)	−0.0120 (14)	−0.0187 (14)
C29	0.072 (2)	0.061 (2)	0.066 (2)	−0.0020 (17)	−0.0222 (17)	−0.0367 (17)
C30	0.087 (3)	0.048 (2)	0.082 (3)	−0.0043 (17)	−0.020 (2)	−0.0406 (18)
C31	0.075 (2)	0.0398 (17)	0.070 (2)	0.0062 (15)	−0.0152 (18)	−0.0256 (16)
C32	0.0427 (14)	0.0333 (14)	0.0412 (15)	0.0030 (11)	−0.0101 (11)	−0.0123 (11)
C33	0.0422 (14)	0.0305 (13)	0.0433 (15)	0.0034 (10)	−0.0170 (12)	−0.0121 (11)
C34	0.0482 (17)	0.0492 (18)	0.077 (2)	0.0091 (14)	−0.0132 (15)	−0.0327 (16)
C35	0.062 (2)	0.0413 (17)	0.087 (2)	0.0153 (15)	−0.0195 (18)	−0.0363 (17)
C36	0.065 (2)	0.0319 (15)	0.077 (2)	0.0068 (13)	−0.0282 (17)	−0.0269 (15)
C37	0.0508 (16)	0.0335 (14)	0.0558 (17)	0.0045 (12)	−0.0239 (14)	−0.0171 (12)
C38	0.0561 (18)	0.0352 (15)	0.068 (2)	−0.0071 (13)	−0.0190 (15)	−0.0113 (14)
C39	0.0523 (17)	0.0407 (17)	0.066 (2)	−0.0086 (13)	−0.0045 (15)	−0.0110 (15)
C40	0.0458 (16)	0.0417 (16)	0.0578 (18)	0.0003 (12)	−0.0085 (13)	−0.0140 (14)
C41	0.0529 (18)	0.054 (2)	0.068 (2)	0.0017 (15)	0.0075 (16)	−0.0197 (17)
C42	0.060 (2)	0.0488 (19)	0.076 (2)	0.0073 (15)	0.0053 (17)	−0.0310 (17)
C43	0.0583 (18)	0.0336 (15)	0.066 (2)	0.0039 (13)	−0.0021 (15)	−0.0236 (14)
C44	0.104 (5)	0.229 (8)	0.127 (5)	0.018 (5)	−0.031 (4)	−0.080 (5)

### Geometric parameters (Å, º)

**Table:**

Mn1—Cl2	2.4185 (9)	C13—C21	1.411 (4)
Mn1—O1	2.266 (2)	C14—C18	1.403 (5)
Mn1—O3	2.257 (2)	C15—H15	0.9300
Mn1—N7	2.173 (2)	C15—C16	1.391 (6)
Mn1—N5	2.302 (2)	C16—H16	0.9300
Mn1—N6	2.230 (2)	C16—C17	1.353 (6)
Cu1—Cl1	2.2775 (9)	C17—H17	0.9300
Cu1—N1	1.998 (2)	C17—C18	1.391 (6)
Cu1—N2	2.090 (2)	C18—C19	1.428 (6)
Cu1—N3	1.997 (2)	C19—H19	0.9300
Cu1—N4	2.174 (3)	C19—C20	1.350 (6)
O1—C25	1.250 (3)	C20—H20	0.9300
O2—C25	1.234 (3)	C20—C21	1.430 (6)
O3—C28	1.261 (4)	C21—C22	1.391 (5)
O4—C28	1.224 (4)	C22—H22	0.9300
O5—H5A	0.8200	C22—C23	1.366 (5)
O5—C44	1.281 (6)	C23—H23	0.9300
N1—C1	1.359 (3)	C23—C24	1.388 (5)
N1—C12	1.330 (4)	C24—H24	0.9300
N2—C2	1.353 (3)	C25—C26	1.518 (4)
N2—C3	1.328 (4)	C26—C31	1.386 (4)
N3—C13	1.352 (4)	C27—C28	1.513 (4)
N3—C24	1.324 (4)	C27—C29	1.379 (4)
N4—C14	1.362 (4)	C29—H29	0.9300
N4—C15	1.330 (5)	C29—C30	1.382 (5)
N7—C26	1.335 (3)	C30—H30	0.9300
N7—C27	1.326 (4)	C30—C31	1.387 (5)
N5—C32	1.352 (3)	C31—H31	0.9300
N5—C43	1.325 (3)	C32—C33	1.441 (4)
N6—C33	1.351 (3)	C32—C40	1.400 (4)
N6—C34	1.328 (4)	C33—C37	1.399 (4)
C1—C2	1.432 (4)	C34—H34	0.9300
C1—C9	1.390 (4)	C34—C35	1.389 (4)
C2—C6	1.402 (4)	C35—H35	0.9300
C3—H3	0.9300	C35—C36	1.343 (5)
C3—C4	1.390 (5)	C36—H36	0.9300
C4—H4	0.9300	C36—C37	1.406 (4)
C4—C5	1.364 (5)	C37—C38	1.425 (4)
C5—H5	0.9300	C38—H38	0.9300
C5—C6	1.399 (4)	C38—C39	1.359 (4)
C6—C7	1.426 (4)	C39—H39	0.9300
C7—H7	0.9300	C39—C40	1.427 (4)
C7—C8	1.349 (4)	C40—C41	1.406 (4)
C8—H8	0.9300	C41—H41	0.9300
C8—C9	1.434 (4)	C41—C42	1.358 (5)
C9—C10	1.394 (4)	C42—H42	0.9300
C10—H10	0.9300	C42—C43	1.381 (4)
C10—C11	1.355 (5)	C43—H43	0.9300
C11—H11	0.9300	C44—H44A	0.9600
C11—C12	1.383 (4)	C44—H44B	0.9600
C12—H12	0.9300	C44—H44C	0.9600
C13—C14	1.436 (5)		
			
O1—Mn1—Cl2	91.88 (6)	N4—C15—C16	122.6 (4)
O1—Mn1—N5	84.75 (8)	C16—C15—H15	118.7
O3—Mn1—Cl2	99.59 (6)	C15—C16—H16	120.2
O3—Mn1—O1	143.98 (7)	C17—C16—C15	119.6 (4)
O3—Mn1—N5	92.69 (8)	C17—C16—H16	120.2
N7—Mn1—Cl2	104.76 (6)	C16—C17—H17	119.9
N7—Mn1—O1	72.07 (8)	C16—C17—C18	120.3 (4)
N7—Mn1—O3	72.00 (8)	C18—C17—H17	119.9
N7—Mn1—N5	89.93 (8)	C14—C18—C19	118.3 (4)
N7—Mn1—N6	155.48 (9)	C17—C18—C14	116.9 (4)
N5—Mn1—Cl2	163.15 (6)	C17—C18—C19	124.8 (4)
N6—Mn1—Cl2	95.19 (6)	C18—C19—H19	119.2
N6—Mn1—O1	122.07 (8)	C20—C19—C18	121.5 (4)
N6—Mn1—O3	90.94 (8)	C20—C19—H19	119.2
N6—Mn1—N5	72.98 (8)	C19—C20—H20	119.1
N1—Cu1—Cl1	94.39 (7)	C19—C20—C21	121.7 (4)
N1—Cu1—N2	80.86 (9)	C21—C20—H20	119.1
N1—Cu1—N4	96.83 (10)	C13—C21—C20	118.2 (4)
N2—Cu1—Cl1	136.84 (7)	C22—C21—C13	117.6 (3)
N2—Cu1—N4	114.64 (10)	C22—C21—C20	124.2 (3)
N3—Cu1—Cl1	93.14 (8)	C21—C22—H22	120.2
N3—Cu1—N1	172.42 (10)	C23—C22—C21	119.6 (3)
N3—Cu1—N2	94.28 (10)	C23—C22—H22	120.2
N3—Cu1—N4	79.84 (11)	C22—C23—H23	120.3
N4—Cu1—Cl1	108.52 (9)	C22—C23—C24	119.5 (4)
C25—O1—Mn1	118.58 (19)	C24—C23—H23	120.3
C28—O3—Mn1	118.40 (19)	N3—C24—C23	122.6 (3)
C44—O5—H5A	109.5	N3—C24—H24	118.7
C1—N1—Cu1	114.18 (18)	C23—C24—H24	118.7
C12—N1—Cu1	127.6 (2)	O1—C25—C26	115.4 (2)
C12—N1—C1	118.2 (2)	O2—C25—O1	126.6 (3)
C2—N2—Cu1	111.40 (17)	O2—C25—C26	118.0 (3)
C3—N2—Cu1	131.1 (2)	N7—C26—C25	114.6 (2)
C3—N2—C2	117.5 (3)	N7—C26—C31	120.7 (3)
C13—N3—Cu1	115.5 (2)	C31—C26—C25	124.7 (3)
C24—N3—Cu1	125.9 (2)	N7—C27—C28	114.2 (2)
C24—N3—C13	118.6 (3)	N7—C27—C29	121.2 (3)
C14—N4—Cu1	110.1 (2)	C29—C27—C28	124.6 (3)
C15—N4—Cu1	132.3 (3)	O3—C28—C27	115.6 (3)
C15—N4—C14	117.5 (3)	O4—C28—O3	125.8 (3)
C26—N7—Mn1	118.99 (19)	O4—C28—C27	118.6 (3)
C27—N7—Mn1	119.67 (18)	C27—C29—H29	120.7
C27—N7—C26	121.3 (2)	C27—C29—C30	118.5 (3)
C32—N5—Mn1	114.83 (17)	C30—C29—H29	120.7
C43—N5—Mn1	127.8 (2)	C29—C30—H30	120.0
C43—N5—C32	117.3 (2)	C29—C30—C31	119.9 (3)
C33—N6—Mn1	116.78 (16)	C31—C30—H30	120.0
C34—N6—Mn1	125.3 (2)	C26—C31—C30	118.4 (3)
C34—N6—C33	117.9 (2)	C26—C31—H31	120.8
N1—C1—C2	116.7 (2)	C30—C31—H31	120.8
N1—C1—C9	122.8 (3)	N5—C32—C33	117.1 (2)
C9—C1—C2	120.5 (2)	N5—C32—C40	123.0 (2)
N2—C2—C1	116.7 (2)	C40—C32—C33	119.9 (2)
N2—C2—C6	123.5 (3)	N6—C33—C32	118.2 (2)
C6—C2—C1	119.8 (2)	N6—C33—C37	122.9 (2)
N2—C3—H3	118.4	C37—C33—C32	118.9 (2)
N2—C3—C4	123.3 (3)	N6—C34—H34	118.8
C4—C3—H3	118.4	N6—C34—C35	122.4 (3)
C3—C4—H4	120.6	C35—C34—H34	118.8
C5—C4—C3	118.9 (3)	C34—C35—H35	119.9
C5—C4—H4	120.6	C36—C35—C34	120.3 (3)
C4—C5—H5	119.9	C36—C35—H35	119.9
C4—C5—C6	120.2 (3)	C35—C36—H36	120.3
C6—C5—H5	119.9	C35—C36—C37	119.4 (3)
C2—C6—C7	118.5 (3)	C37—C36—H36	120.3
C5—C6—C2	116.6 (3)	C33—C37—C36	117.2 (3)
C5—C6—C7	124.9 (3)	C33—C37—C38	120.2 (3)
C6—C7—H7	119.0	C36—C37—C38	122.6 (3)
C8—C7—C6	121.9 (3)	C37—C38—H38	119.7
C8—C7—H7	119.0	C39—C38—C37	120.6 (3)
C7—C8—H8	119.7	C39—C38—H38	119.7
C7—C8—C9	120.6 (3)	C38—C39—H39	119.6
C9—C8—H8	119.7	C38—C39—C40	120.8 (3)
C1—C9—C8	118.9 (3)	C40—C39—H39	119.6
C1—C9—C10	117.2 (3)	C32—C40—C39	119.5 (3)
C10—C9—C8	123.9 (3)	C32—C40—C41	117.2 (3)
C9—C10—H10	120.1	C41—C40—C39	123.2 (3)
C11—C10—C9	119.8 (3)	C40—C41—H41	120.4
C11—C10—H10	120.1	C42—C41—C40	119.3 (3)
C10—C11—H11	120.0	C42—C41—H41	120.4
C10—C11—C12	120.0 (3)	C41—C42—H42	120.3
C12—C11—H11	120.0	C41—C42—C43	119.4 (3)
N1—C12—C11	122.0 (3)	C43—C42—H42	120.3
N1—C12—H12	119.0	N5—C43—C42	123.7 (3)
C11—C12—H12	119.0	N5—C43—H43	118.2
N3—C13—C14	118.0 (3)	C42—C43—H43	118.2
N3—C13—C21	122.1 (3)	O5—C44—H44A	109.5
C21—C13—C14	119.8 (3)	O5—C44—H44B	109.5
N4—C14—C13	116.5 (3)	O5—C44—H44C	109.5
N4—C14—C18	123.1 (3)	H44A—C44—H44B	109.5
C18—C14—C13	120.4 (3)	H44A—C44—H44C	109.5
N4—C15—H15	118.7	H44B—C44—H44C	109.5

### Hydrogen-bond geometry (Å, º)

**Table:**

*D*—H···*A*	*D*—H	H···*A*	*D*···*A*	*D*—H···*A*
C22—H22···Cl2^i^	0.93	2.87	3.683 (4)	147
C34—H34···Cl2	0.93	2.88	3.492 (3)	124
C30—H30···Cl2^ii^	0.93	2.87	3.751 (3)	158
C44—H44*C*···Cl1^iii^	0.96	2.86	3.574 (6)	132
O5—H5*A*···O2	0.82	2.03	2.798 (5)	155
C20—H20···O3	0.93	2.45	3.337 (4)	159

Symmetry codes: (i) −*x*, −*y*+1, −*z*+1; (ii) −*x*, −*y*, −*z*+1; (iii) *x*, *y*−1, *z*+1.
